# ASO Author Reflections: Association of COVID-19 Pandemic with Colorectal Cancer Screening: Impact of Race/Ethnicity and Social Vulnerability

**DOI:** 10.1245/s10434-024-15097-z

**Published:** 2024-02-21

**Authors:** Muhammad Muntazir Mehdi Khan, Muhammad Musaab Munir, Timothy M. Pawlik

**Affiliations:** Department of Surgery, The Ohio State University Wexner Medical Center and James Comprehensive Cancer Center, Columbus, OH USA

## Past

Colorectal cancer (CRC) is the third most common cancer in the United States. Given the efficacy of screening for early detection,^1^ the U.S. Preventive Services Task Force recommends screening for adults aged 45–74 years.^2^ The COVID-19 pandemic disrupted healthcare delivery, including cancer screening.^3^ Various policies were implemented to mitigate COVID-19 transmission, which affected primary healthcare services and altered cancer screening practices.^4^ Despite the easing of restrictions, screening for cancers did not fully recover post-pandemic with vulnerable populations possibly at higher risk for suboptimal screening.^5^ To date, the impact of the pandemic on CRC screening, particularly among socially vulnerable individuals, remains poorly defined. We sought to characterize CRC screening utilization during the pandemic, as well as post-pandemic return of screening practices relative to county-level SVI and race/ethnicity in a nationally representative cohort.^6^

## Present

Among 10,503,180 individuals continuously enrolled in Medicare with no previous diagnosis of CRC, 1,362,457 (12.97%) underwent CRC screening between 2019–2021. With the COVID-19 pandemic, CRC screening decreased markedly across the United States (median monthly screening: pre-pandemic *n* = 76,444 vs. pandemic *n* = 60,826; median Δ*n* = 15,618; *p* < 0.001). One-year post-pandemic, overall CRC screening utilization generally rebounded to pre-COVID-19 levels (monthly median screening volumes: pandemic, *n* = 60,826, vs. post-pandemic, *n* = 74,170; median Δ*n* = 13,344; *p* < 0.001). Individuals residing in counties with the highest SVI experienced the largest decline in CRC screening odds relative to individuals residing in low SVI counties (reference: low SVI; pre-pandemic: high SVI OR 0.85; pandemic: high SVI OR 0.81; post-pandemic: high SVI OR 0.85; all *p* < 0.001).

## Future

Cancer screening can be impacted by social determinants of health, including race/ethnicity and social vulnerability. Perhaps not surprising, there was a much lower utilization of CRC during the COVID-19 pandemic. Of more interest was the finding that screening did not rebound to pre-pandemic levels equally among the U.S. population; rather, racial/ethnic minority patients, as well as individuals living in vulnerable areas, experienced a persistent lower chance to have CRC screening post-pandemic. These data serve to highlight how social inequities not only differentially impacted healthcare delivery during the pandemic, but also how these disparities persisted post-pandemic. Focused efforts to coordinate healthcare delivery systems, as well as increase utilization of telehealth services/mobile screening services are needed to address cancer screening disparities.
